# An interpretable deep learning framework based on TabNet-Cox for risk stratification and prognostic assessment in hepatocellular carcinoma immunotherapy

**DOI:** 10.3389/fimmu.2026.1751829

**Published:** 2026-02-11

**Authors:** Wei Dong, Cheng Qian

**Affiliations:** 1 Department of Hepatobiliary and Pancreatic Surgery, No. 150 Haping Road Harbin Medical University Cancer Hospital, Harbin, Heilongjiang, China; 2 Department of Breast Surgery, No. 150 Haping Road Harbin Medical University Cancer Hospital, Harbin, Heilongjiang, China

**Keywords:** deep learning, hepatocellular carcinoma, immune checkpoint inhibitors, risk stratification, survival prediction

## Abstract

**Objective:**

Therapeutic outcomes after immune checkpoint inhibitors (ICIs) in hepatocellular carcinoma (HCC) are highly heterogeneous. Accurate prognostic assessment is essential for risk stratification and clinical management. This study aimed to develop and validate an interpretable deep-learning survival model, TabNet-Cox, for predicting overall survival (OS) in ICI-treated HCC patients.

**Methods:**

A total of 453 consecutive HCC patients treated with ICIs at Harbin Medical University Cancer Hospital between January 2018 and December 2023 were retrospectively enrolled and randomly assigned to a training cohort (n = 339) and an internal validation cohort (n = 114). An independent external validation cohort of 105 patients was collected from the Second Affiliated Hospital of Harbin Medical University under the same inclusion criteria. Baseline demographic variables, tumor characteristics, pretreatment management categories (surgery, locoregional therapy, or none), and laboratory parameters were used to develop TabNet-Cox. Model performance was assessed under a repeated 5-fold cross-validation protocol and further evaluated in the internal and external cohorts using the concordance index (C-index), AUC, and Brier score. SHapley Additive exPlanations (SHAP) and unsupervised clustering were applied for interpretability and phenotype exploration. Clinical utility was examined using decision curve analysis (DCA) with BCLC stage as the reference.

**Results:**

TabNet-Cox showed the best overall performance among the survival models compared, achieving a C-index of 0.79 and an AUC of 0.81 with the lowest Brier score (0.059) in the development setting. In the external validation cohort, TabNet-Cox demonstrated stable discriminative performance, with well-defined ROC curves and good calibration. Using the prespecified risk cut-off, the model effectively stratified patients into distinct risk groups, yielding significantly separated Kaplan–Meier survival curves (P < 0.001). SHAP analysis highlighted AFP, GGT, and LDH as major risk contributors, whereas albumin and lymphocyte count were protective. Unsupervised clustering within high-risk patients suggested two patterns, a tumor burden-dominant phenotype and a liver dysfunction-dominant phenotype, which should be interpreted as hypothesis-generating.

**Conclusion:**

TabNet-Cox provides an accurate and interpretable framework for OS prediction and risk stratification in ICI-treated HCC using routinely available baseline variables. Its performance was supported by resampling-based evaluation and independent external validation, supporting its potential value for individualized prognostic assessment.

## 1 Introduction

Primary liver cancer is the sixth most common cancer and the third leading cause of cancer-related death worldwide, with hepatocellular carcinoma (HCC) accounting for most cases (1). While surgical resection remains the curative standard for early-stage disease, a significant proportion of patients are diagnosed at advanced stages or experience postoperative recurrence (2). In recent years, immune checkpoint inhibitors (ICIs), such as those targeting PD-1/PD-L1, have revolutionized the treatment landscape of HCC, offering durable survival benefits (3, 4). However, the therapeutic response to ICIs is highly heterogeneous; only a subset of patients achieves objective response or long-term survival, while others may suffer from hyper-progression or immune-related adverse events (5). Therefore, accurate risk stratification and prognostic prediction are urgently needed to optimize personalized management and avoid futile treatments.

Prognostic assessment in HCC is particularly challenging due to the complex interplay between tumor burden and underlying liver dysfunction. Traditional staging systems, such as the Barcelona Clinic Liver Cancer (BCLC) staging, primarily rely on categorical variables and may not fully capture the biological heterogeneity of patients receiving immunotherapy (6). Similarly, conventional statistical methods like the Cox proportional hazards model assume linear relationships between covariates and risk, which limits their ability to uncover complex, non-linear interactions within high-dimensional clinical data (7). Although individual biomarkers (e.g., AFP) are widely used, their standalone predictive power is often insufficient for precise individual survival prediction (8).

With the advent of artificial intelligence, machine learning (ML) and deep learning (DL) have demonstrated superior performance in survival analysis compared to traditional approaches (9). Algorithms such as Random Survival Forests (RSF) and DeepSurv have shown promise in handling complex datasets (10). However, a critical barrier to the clinical adoption of most DL models is their “black-box” nature—they often lack interpretability, leaving clinicians unable to understand the logic behind specific risk predictions (11). In clinical practice, trust and transparency are as crucial as predictive accuracy (12). Consequently, there is a growing demand for interpretable deep learning frameworks that can deliver high-performance predictions while providing biological insights into the decision-making process.

To address these challenges, we introduced TabNet, a novel deep learning architecture designed specifically for tabular data, into the survival analysis of HCC (13). TabNet employs a sequential attention mechanism to perform instance-wise feature selection, effectively combining the interpretability of tree-based methods with the representation learning capability of deep neural networks (14). By integrating the TabNet architecture with the Cox loss function, we developed a TabNet-Cox model to predict survival conditions in HCC patients treated with ICIs. In this study, we aimed to construct and validate this interpretable model using multi-dimensional clinical and laboratory parameters. Beyond merely improving prediction accuracy, we focused on elucidating the model’s decision logic through SHapley Additive exPlanations (SHAP) values and attention masks, thereby identifying high-risk clinical profiles and facilitating biologically plausible risk stratification for HCC patients undergoing immunotherapy (15).

## 2 Patients and methods

### 2.1 Patients

This retrospective study was conducted at Harbin Medical University Cancer Hospital, enrolling patients diagnosed with HCC who underwent ICIs therapy between January 2018 and December 2023. The diagnosis of HCC was established based on histopathological examination or non-invasive radiologic criteria (CT or MRI) according to the guidelines of the Chinese Society of Clinical Oncology (CSCO). To ensure the homogeneity of the study population and the quality of data for model construction, we applied rigorous eligibility criteria.

The inclusion criteria were as follows: (1) Age ≥ 18 years; (2) Confirmed diagnosis of HCC; (3) Receipt of ICI treatment (PD-1 or PD-L1 inhibitors), either as monotherapy or in combination with targeted therapy or locoregional treatments; (4) Availability of complete baseline clinical characteristics and laboratory parameters (including liver function, renal function, coagulation profile, and tumor markers) prior to the initial ICI administration; (5) Availability of definitive follow-up data and survival outcomes. Patients were excluded from the study if they met any of the following criteria: (1) Diagnosed with combined other concurrent primary malignancies; (2) History of liver transplantation or severe autoimmune diseases excluding viral hepatitis; (3) Absence of key pretreatment serological indicators essential for the TabNet model; (4) Loss to follow-up or unknown survival status; (5) Clinical evidence of active infection requiring systemic antibiotic therapy at the time of baseline assessment, which could interfere with inflammatory biomarkers.

Based on these criteria, a total of 453 eligible patients were finally identified. The cohort was randomly partitioned into a training cohort (n = 339) and a validation cohort (n = 114) at a 3:1 ratio using a random number table method, in which each patient was assigned a unique study ID and allocated according to the generated random number. In addition, 105 patients were collected from the Second Affiliated Hospital of Harbin Medical University during the same study period under the same eligibility criteria and were used exclusively as an external validation cohort. This study strictly adhered to the ethical principles of the Declaration of Helsinki. The research protocol was reviewed and approved by the Ethics Committee of Harbin Medical University Cancer Hospital (Approval No. ALTN-AK105-III-06).

### 2.2 Treatment protocols and data acquisition

Before initiation of ICI–based therapy, some patients had received prior treatments, which mainly included surgery and locoregional therapies, as clinically indicated. Locoregional therapies comprised transarterial chemoembolization (TACE), hepatic arterial infusion chemotherapy (HAIC), radiotherapy, or ablation. All enrolled patients subsequently received combined targeted therapy and immunotherapy and completed a minimum of four treatment cycles. The treatment regimens primarily consisted of two protocols: (1) the “Atezo+Bev” regimen, comprising intravenous atezolizumab (1200 mg) plus bevacizumab (15 mg/kg) administered every three weeks; and (2) a clinical trial protocol (Registration No. CTR20211710), involving intravenous camrelizumab (200 mg for body weight ≥50 kg; 3 mg/kg for <50 kg) every two weeks, combined with daily oral apatinib (250 mg).

To ensure the data reflected the patient’s status before therapeutic intervention, all baseline demographic, clinicopathological, and laboratory data were retrospectively extracted from the electronic medical records system prior to the initiation of the treatments. To comprehensively assess the pre-treatment physiological profile, we collected a multidimensional set of variables, including demographics (age, sex, BMI, lifestyle factors), tumor characteristics (tumor size, number, and BCLC stage), and treatment history. Additionally, a broad panel of baseline laboratory parameters was obtained, covering liver and renal function tests, hematological and inflammatory markers, coagulation profiles, tumor biomarkers, and immunoglobulin levels. To provide a standardized assessment of baseline liver function, we additionally calculated the albumin–bilirubin (ALBI) score using the following formula: ALBI = 0.66 × log_10_(bilirubin [μmol/L]) − 0.085 × albumin [g/L]. The ALBI score is an objective, laboratory-based index derived solely from total bilirubin and albumin, enabling quantitative assessment of hepatic functional reserve without reliance on subjective clinical components, and is widely used for prognostic stratification in hepatocellular carcinoma (16, 17). The primary endpoint was overall survival (OS), defined as the time interval from the first date of ICI administration to death from any cause or the last follow-up. Survival data were updated via outpatient reviews and telephone interviews, with patients alive at the last contact censored. Prior to model construction, continuous variables were standardized using Z-score normalization, while categorical variables were numerically encoded.

### 2.3 TabNet-Cox model

To overcome the inherent limitations of conventional Cox proportional hazards regression in capturing high-dimensional, non-linear feature interactions while addressing the interpretability issues of standard deep neural networks, we employed the TabNet-Cox architecture. TabNet is a deep neural network specifically optimized for tabular data through a sequential attention mechanism that enables instance-wise feature selection (18). The model operates via a multi-step decision process, where at each decision step, an Attentive Transformer learns a soft mask matrix to selectively focus on the most informative features based on the context of previous steps (19). The masked features are then processed by a Feature Transformer to extract high-level representations. By aggregating these representations across all decision steps, TabNet effectively combines the interpretability of tree-based decision processes with the expressive power of deep learning (20).

In the survival analysis setting, we modified the final prediction layer of TabNet to output a single scalar log-risk score, denoted as ℎ_θ_(*x*), where *x* represents the patient feature vector and θ the trainable parameters. Under the proportional hazard’s assumption, the individual hazard function is expressed as: λ(*t*|*x*) = λ_0_(*t*)exp(ℎ_θ_(*x*)), where λ_0_(*t*) is the baseline hazard function. This allows the model to estimate relative risks of mortality across patients based on their clinical profiles.

Model training was conducted by minimizing the negative log-partial likelihood of the Cox model, combined with an entropy-based sparsity penalty applied to the attention masks to promote interpretability and prevent overfitting. For a cohort of *N* patients, the overall objective function is given by:


$$\begin{array}{c} L\left(\theta \right)=-\sum_{i:E_{i}=1}\left[h_{\theta }\left(x_{i}\right)-\log\sum_{j\in R\left(t_{i}\right)}\exp\left(h_{\theta }\left(x_{j}\right)\right)\right]\\ +\lambda_{sparse}\frac{1}{N_{steps}\cdot B}\sum_{i=1}^{N_{steps}}\sum_{b=1}^{B}\sum_{j=1}^{D}\left[-M_{b,j}\left[i\right]\log M_{b,j}\left[i\right]\right] \end{array}$$

Here, *E_i_* is the event indicator (1 for death, 0 for censored), and *R*(*t_i_*) is the risk set of individuals still under observation at time *t_i_*. The second term imposes entropy-based sparsity regularization on the attention masks *M*, controlled by the hyperparameter λ_sparse_. This term encourages each decision step to focus on a concise subset of features, thereby improving both model interpretability and generalization. Through this integration of Cox partial likelihood and sparse attentional feature selection, the TabNet-Cox model achieves accurate survival prediction while maintaining transparency and theoretical consistency with classical survival analysis frameworks.

### 2.4 Model comparison and evaluation metrics

To place the performance of TabNet-Cox in an appropriate methodological context, we compared it with several commonly used survival modeling approaches representing different methodological paradigms. These included traditional statistical models (Cox proportional hazards regression, CoxPH), tree-based ensemble methods (random survival forests, RSF), boosting-based survival models (gradient-boosting survival analysis, GBSA), distance-based methods (k-nearest-neighbor survival, KNN survival), and neural network–based approaches (DeepSurv). CoxPH is a conventional proportional hazards model that assumes a log-linear relationship between covariates and the hazard function and serves as a widely accepted statistical benchmark in clinical prognostic studies (21). RSF is an ensemble method based on survival trees, specifically adapted for censored data, and can capture non-linear effects and complex variable interactions (22). GBSA refers to survival models constructed using gradient boosting, in which multiple weak learners are combined in an additive manner to flexibly model non-linear relationships (23). KNN survival is a non-parametric, distance-based approach that estimates survival outcomes by borrowing information from patients with similar baseline characteristics (24). DeepSurv is a neural network–based extension of Cox regression that replaces the linear predictor with a non-linear function learned from the data, allowing for more flexible risk modeling while retaining the Cox partial likelihood framework (25). To ensure comparability across methods, all models were trained using the same set of baseline features and evaluated under an identical resampling-based evaluation protocol and performance metrics.

To ensure a fair and robust comparison across modeling paradigms, all models were evaluated under the same resampling-based protocol. Specifically, we performed repeated 5-fold cross-validation with 10 repeats using different random seeds within the training cohort, and summarized performance by the mean concordance index (C-index) and AUC across runs. For TabNet-Cox, hyperparameters were optimized within the training data using grid search under the same resampling protocol, and the final configuration was fixed for all subsequent analyses (**Supplementary Table 1**). After model selection, the final models were retrained on the full training cohort and evaluated once on the held-out internal validation cohort and the external cohort without refitting. Model training used the Adam optimizer with early stopping. Because CoxPH is more sensitive to multicollinearity among predictors, we implemented a standardized Cox modeling procedure. All candidate variables were entered as continuous terms in univariable Cox regression, and variables with *P < 0.05* were further assessed for collinearity using the variance inflation factor (VIF) and tolerance statistics. Variables meeting the collinearity criteria were jointly included in the multivariable Cox model, and the ALBI score was additionally incorporated to provide a standardized measure of baseline liver function and improve comparability with established clinical indices. The proportional hazards assumption was assessed for the fitted Cox model using Schoenfeld residuals.

Model discrimination was assessed using C-index and the area under the receiver operating characteristic curve (AUC) computed from model-predicted risk scores under the same evaluation setting. Prediction error was summarized using the Brier score and the integrated Brier score (IBS) under right censoring over the evaluation horizon, where lower values indicate better accuracy. Model stability was quantified by the stability index (σ), defined as the standard deviation of the C-index across repeated resampling runs with the same evaluation protocol, with lower σ indicating more reproducible performance. Absolute prediction errors were additionally visualized using 3D error surface plots to facilitate intuitive comparison of error patterns across models.

### 2.5 Statistical analysis

Statistical analyses were performed using Python (version 3.8) and R software (version 4.2.0). Continuous variables were assessed for normality using the Kolmogorov–Smirnov test. Normally distributed data were expressed as mean ± standard deviation (SD) and compared using Student’s t-test. Non-normally distributed data were presented as median with interquartile range (IQR) and compared using the Mann–Whitney U test. Categorical variables were reported as frequencies and percentages, with differences between groups evaluated using the Chi-square test or Fisher’s exact test. Survival curves were estimated using the Kaplan–Meier method and compared using the log-rank test.

An optimal cut-off value for the predicted risk score was determined to stratify patients into high- and low-risk groups. Cox regression analyses were performed to identify independent prognostic factors, with results expressed as hazard ratios (HRs) and 95% confidence intervals (CIs). Subgroup analyses based on independent prognostic factors (surgery status and BCLC stage) were conducted to assess robustness. Model interpretability was evaluated using SHAP values. Feature correlations were analyzed using Pearson correlation coefficients, and high-risk phenotypes were identified using unsupervised hierarchical clustering analysis. Clinical utility was assessed using decision curve analysis (DCA), and calibration curves were plotted to examine agreement between predicted and observed survival probabilities. All statistical tests were two-sided, and *P < 0.05* was considered statistically significant.

## 3 Results

### 3.1 Patients characteristic

A total of 453 patients with HCC were included, comprising 339 in the training cohort and 114 in the validation cohort (**Table 1**). The baseline clinical characteristics were generally comparable between the two cohorts. Most patients were male (81.7% *vs*. 83.3%, *P = 0.778*), with a mean age of 57.5 ± 9.2 years and 57.1 ± 8.9 years, respectively (*P = 0.640*). The mean BMI was 23.3 ± 3.5 kg/m² and 23.5 ± 3.8 kg/m² in the two cohorts (*P = 0.545*). There were no significant differences in smoking or drinking status between groups. Regarding disease stage, 56.6% and 55.3% of patients were classified as BCLC stage C in the training and validation cohorts, respectively. These findings indicate that most patients had advanced-stage HCC, because ICIs are currently used as first-line therapy only for advanced-stage HCC, resulting in a predominantly composed of advanced cases.

**Table 1:** Patients characteristic.

Items	Training (n=339)	Validation (n=114)	*P*
Sex			0.652
Male	277 (81.7%)	95 (83.3%)	
Female	62 (18.3%)	19 (16.7%)	
Age (years)	57.53 ± 9.20	57.07 ± 8.89	0.670
BMI (Kg/m²)	23.28 ± 3.49	23.53 ± 3.76	0.333
ALBI	-2.41 ± 0.44	-2.63 ± 0.38	0.546
Smoking			0.381
Yes	69 (20.4%)	24 (21.1%)	
No	270 (79.6%)	90 (78.9%)	
Drinking			0.515
Yes	41 (12.1%)	16 (14.0%)	
No	298 (87.9%)	98 (86.0%)	
ABO blood type			0.634
A	92 (27.1%)	34 (29.8%)	
B	104 (30.7%)	31 (27.2%)	
AB	53 (15.6%)	13 (11.4%)	
O	90 (26.5%)	36 (31.6%)	
Treatment			0.449
Surgery	112 (33.0%)	41 (36.0%)	
Locoregional therapy	199 (58.7%)	64 (56.1%)	
None	28 (8.3%)	9 (7.9%)	
Tumor number			0.713
Single	137 (40.4%)	49 (43.0%)	
Multiple	202 (59.6%)	65 (57.0%)	
Tumor size			0.475
<5 cm	59 (17.4%)	25 (21.9%)	
≥5 cm	280 (82.6%)	89 (78.1%)	
Liver cirrhosis			0.742
Yes	100 (29.5%)	35 (30.7%)	
No	239 (70.5%)	79 (69.3%)	
BCLC stage			0.814
A + B	147 (43.4%)	51 (44.7%)	
C	192 (56.6%)	63 (55.3%)	

### 3.2 Variables included in the model

The variables included in the model covered a comprehensive range of clinical and laboratory parameters, encompassing liver and renal function tests, metabolic and coagulation profiles, tumor biomarkers, and immunoglobulin levels. These indicators were selected to capture both the physiological and pathological status of patients with hepatocellular carcinoma, providing a multidimensional representation of their systemic condition. No significant differences were found between the training and validation cohorts across any of these parameters (all *P > 0.05*), suggesting that the two cohorts were well-balanced and comparable at baseline (**Table 2**).

**Table 2:** Characteristics of modeling variables.

Items	Training cohort (n=339)	Validation cohort (n=114)	*P*
ALT (U/L), median (IQR)	34.00 (21.50–49.50)	28.00 (22.00–44.75)	0.729
AST (U/L), median (IQR)	48.00 (31.50–83.00)	42.50 (30.00–86.25)	0.422
γ-GGT (U/L), median (IQR)	100.00 (50.00–249.00)	87.00 (41.00–188.25)	0.112
ALP (U/L), median (IQR)	123.00 (92.00–204.00)	109.50 (89.00–190.50)	0.347
TBIL (μmol/L), median (IQR)	20.23 (14.50–32.00)	19.10 (14.95–33.83)	0.942
DBIL (μmol/L), median (IQR)	5.20 (3.20–10.50)	4.48 (3.20–10.08)	0.633
IDBIL (μmol/L), median (IQR)	15.30 (11.10–22.20)	15.30 (11.57–24.10)	0.446
TP (g/L), median (IQR)	72.00 (68.05–78.10)	72.00 (68.17–75.78)	0.687
ALB (g/L), mean (SD)	37.48 ± 5.59	38.29 ± 5.20	0.158
GLOB (g/L), mean (SD)	34.75 ± 8.01	33.76 ± 6.18	0.176
PALB (mg/L), median (IQR)	145.00 (101.00–199.00)	152.00 (101.00–211.00)	0.365
Urea (mmol/L), median (IQR)	5.40 (4.40–7.00)	5.25 (4.50–6.40)	0.620
CREA (μmol/L)	72.92 ± 15.24	74.61 ± 14.47	0.287
UA (μmol/L), median (IQR)	297.00 (251.00–359.00)	313.00 (231.00–375.00)	0.730
LDH (U/L), median (IQR)	227.00 (184.50–297.00)	224.00 (184.25–273.00)	0.485
Glu (mmol/L), mean (SD)	5.56 ± 1.54	5.75 ± 1.57	0.249
WBC (10^9^/L), median (IQR)	6.24 (4.82–7.69)	5.57 (4.89–7.25)	0.150
LYM (10^9^/L), median (IQR)	1.71 (1.36–2.13)	1.71 (1.29–2.04)	0.885
NEU (10^9^/L), median (IQR)	3.81 (2.87–5.23)	3.83 (2.76–5.54)	0.613
MON (10^9^/L), median (IQR)	0.46 (0.32–0.62)	0.51 (0.34–0.62)	0.419
RBC (10^9^/L), mean (SD)	4.42 ± 0.72	4.52 ± 0.71	0.211
HGB (10^9^/L), mean (SD)	137.43 ± 23.91	140.52 ± 23.35	0.226
HCT (10^9^/L), mean (SD)	41.35 ± 6.83	42.34 ± 6.04	0.144
PLT (10^9^/L), median (IQR)	228.00 (178.50–306.00)	250.00 (198.50–306.00)	0.081
PT (s), mean (SD)	12.62 ± 1.78	12.42 ± 1.25	0.189
INR, median (IQR)	1.07 (1.02–1.15)	1.06 (1.01–1.13)	0.386
Fbg (g/L), median (IQR)	2.98 (2.41–3.93)	2.85 (2.41–3.77)	0.634
TT (s), mean (SD)	16.86 ± 1.67	16.91 ± 1.30	0.764
CEA (ng/mL), median (IQR)	2.38 (1.73–3.49)	2.53 (1.59–3.97)	0.896
AFP (ng/mL), median (IQR)	151.40 (5.89–1210.00)	137.20 (5.71–1210.00)	0.971
CA199 (U/mL), median (IQR)	24.35 (10.17–52.28)	18.93 (9.86–47.15)	0.181
IgA (g/L), median (IQR)	2.12 (1.63–2.88)	2.42 (1.67–3.00)	0.083
IgG (g/L), mean (SD)	10.61 ± 2.82	10.99 ± 3.20	0.263
IgM (g/L), median (IQR)	0.87 (0.63–1.22)	0.82 (0.57–1.14)	0.139

### 3.3 Model construction

Six survival prediction models (CoxPH, RSF, GBSA, KNN-Survival, DeepSurv, and TabNet-Cox) were developed and evaluated using mean C-index, AUC, Brier score, IBS, and stability (σ) (**Table 3**). TabNet-Cox performed best overall, achieving the highest discrimination (mean C-index 0.80; AUC 0.81), the lowest prediction error (Brier score 0.059; IBS 0.069), and the greatest stability (σ = 0.014). DeepSurv showed the next-best performance (C-index 0.73; AUC 0.77), whereas CoxPH, GBSA, and KNN-Survival demonstrated similar discrimination (C-index 0.70–0.71; AUC 0.75–0.76). RSF yielded slightly lower discrimination (C-index 0.70; AUC 0.73) with higher error metrics.

**Table 3:** Performance comparison of six survival prediction models.

Model	Mean C-index	AUC	Brier Score	IBS	Stability (σ)
RSF	0.70	0.73	0.076	0.085	0.021
GBSA	0.71	0.75	0.072	0.081	0.02
CoxPH	0.70	0.75	0.071	0.081	0.019
KNN–Survival	0.71	0.76	0.07	0.078	0.019
DeepSurv	0.73	0.77	0.065	0.074	0.017
TabNet-Cox	0.80	0.81	0.059	0.069	0.014

The Brier score and IBS measure prediction error under right censoring. Lower values indicate better accuracy, with 0 denoting perfect prediction. The stability index (σ) reflects performance variability across repeated resampling runs. Lower σ indicates more stable and reproducible performance.

**Figure 1:**
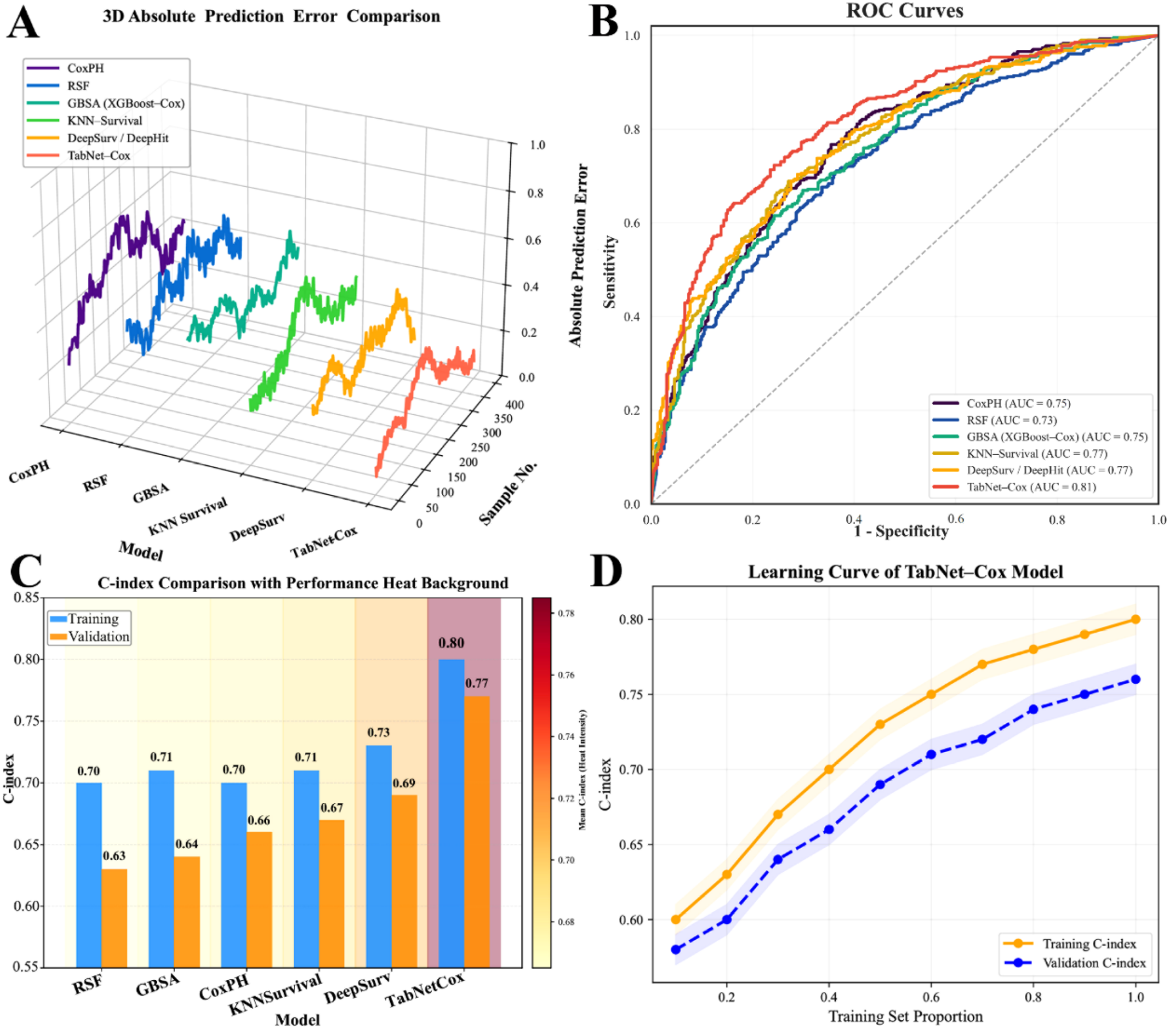
Performance comparison of survival prediction models. **(A)** 3D visualization of absolute prediction errors across different models. **(B)** ROC curves showing model discrimination ability. **(C)** Comparison of C-index between the training and validation cohorts. **(D)** Learning curves of the TabNet–Cox model demonstrating performance stability across varying training set sizes.

Visualization analyses further confirmed the superior performance of TabNet-Cox. In the 3D absolute prediction error plot (**Figure 1A**), error fluctuations decreased progressively from CoxPH to TabNet-Cox, which demonstrated the smallest and smoothest prediction deviations. The ROC curves (**Figure 1B**) showed that TabNet–Cox achieved the highest discrimination power, with its curve closest to the upper-left corner, reflecting stronger sensitivity and specificity. The C-index comparison (**Figure 1C**) revealed consistent performance between training and validation cohorts, with TabNet-Cox maintaining the highest values (0.80 and 0.77, respectively). Furthermore, the learning curve analysis (**Figure 1D**) illustrated a steady upward trend in performance as the training data size increased. The narrow and consistent gap between the training and validation curves indicated that the model achieved good convergence and possessed strong generalization capabilities without significant overfitting. Quantitative and visual evaluations together confirmed that TabNet-Cox outperformed conventional and machine learning-based survival models in terms of discrimination, calibration, and stability.

### 3.4 Model interpretability and feature analysis

**Figure 2:**
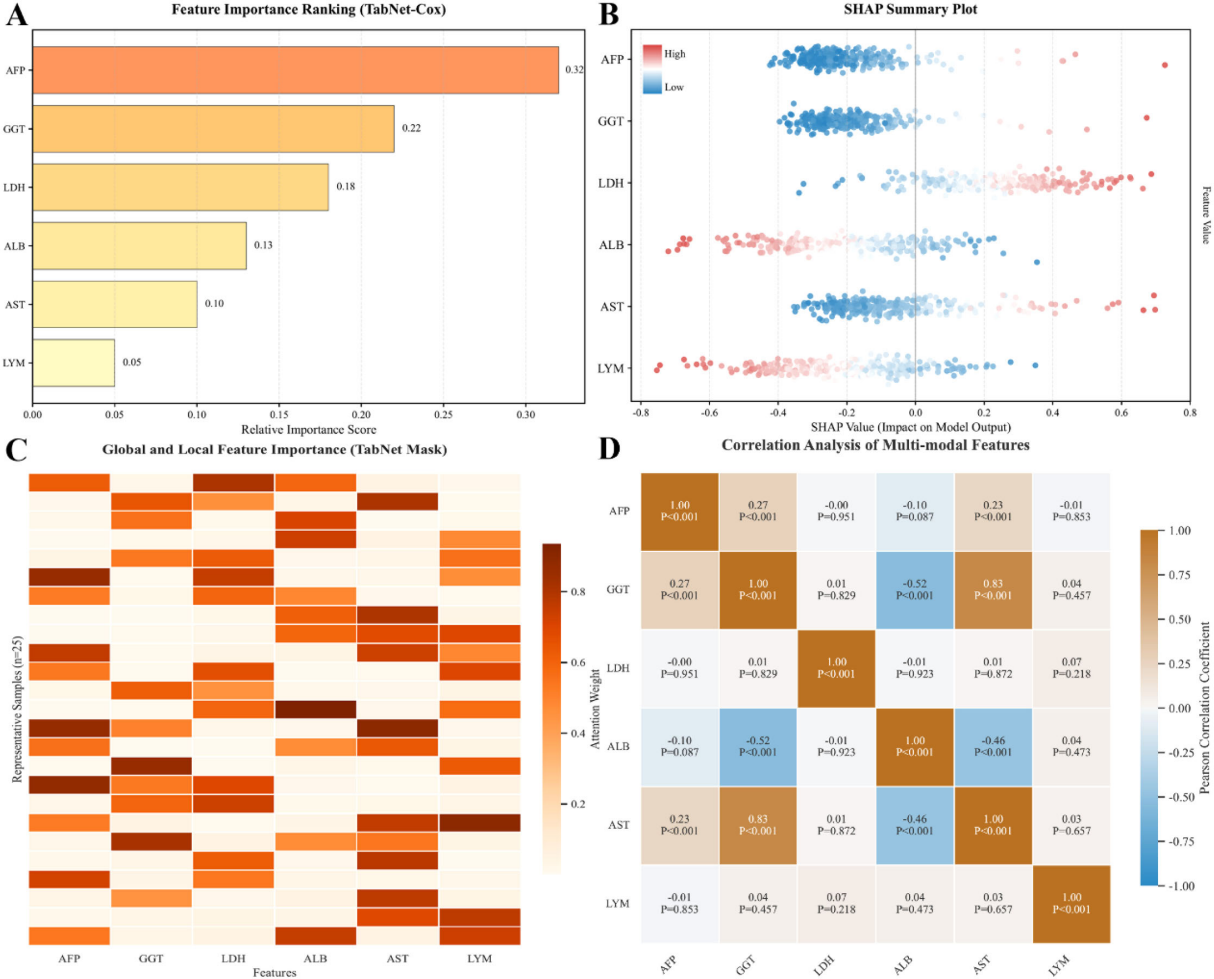
Model interpretability and feature analysis. **(A)** Global feature importance ranking of the top clinical variables. **(B)** SHAP summary plot illustrating the directional impact of features on predicted mortality risk. **(C)** Visualization of TabNet attention masks demonstrating instance-wise feature selection patterns. **(D)** Correlation heatmap showing relationships among the multi-modal clinical features.

To ensure clinical reliability, we analyzed the interpretability of the TabNet-Cox model. The global feature importance ranking (**Figure 2A**) identified AFP as the primary prognostic factor, followed by GGT and LDH. The SHAP summary plot (**Figure 2B**) further clarified the directional impact of these features. High levels of AFP, GGT, LDH, and AST were associated with positive SHAP values, indicating increased mortality risk. In contrast, higher Albumin and Lymphocyte counts corresponded to negative SHAP values, serving as protective factors.

Beyond global trends, the attention mask visualization (**Figure 2C**) demonstrated instance-wise feature selection. The heatmap showed dynamic attention weights, where the model prioritized tumor markers for some patients and liver function or immune indicators for others. Furthermore, correlation analysis (**Figure 2D**) highlighted the relationships between features, such as the strong positive correlation between AST and GGT (*r = 0.83, P < 0.001*) and the negative correlation between Albumin and GGT (*r = -0.52, P < 0.001*). These results indicate that the model effectively integrates multi-modal features despite their intrinsic correlations.

### 3.5 Risk stratification and identification of high-risk clinical profiles

**Figure 3:**
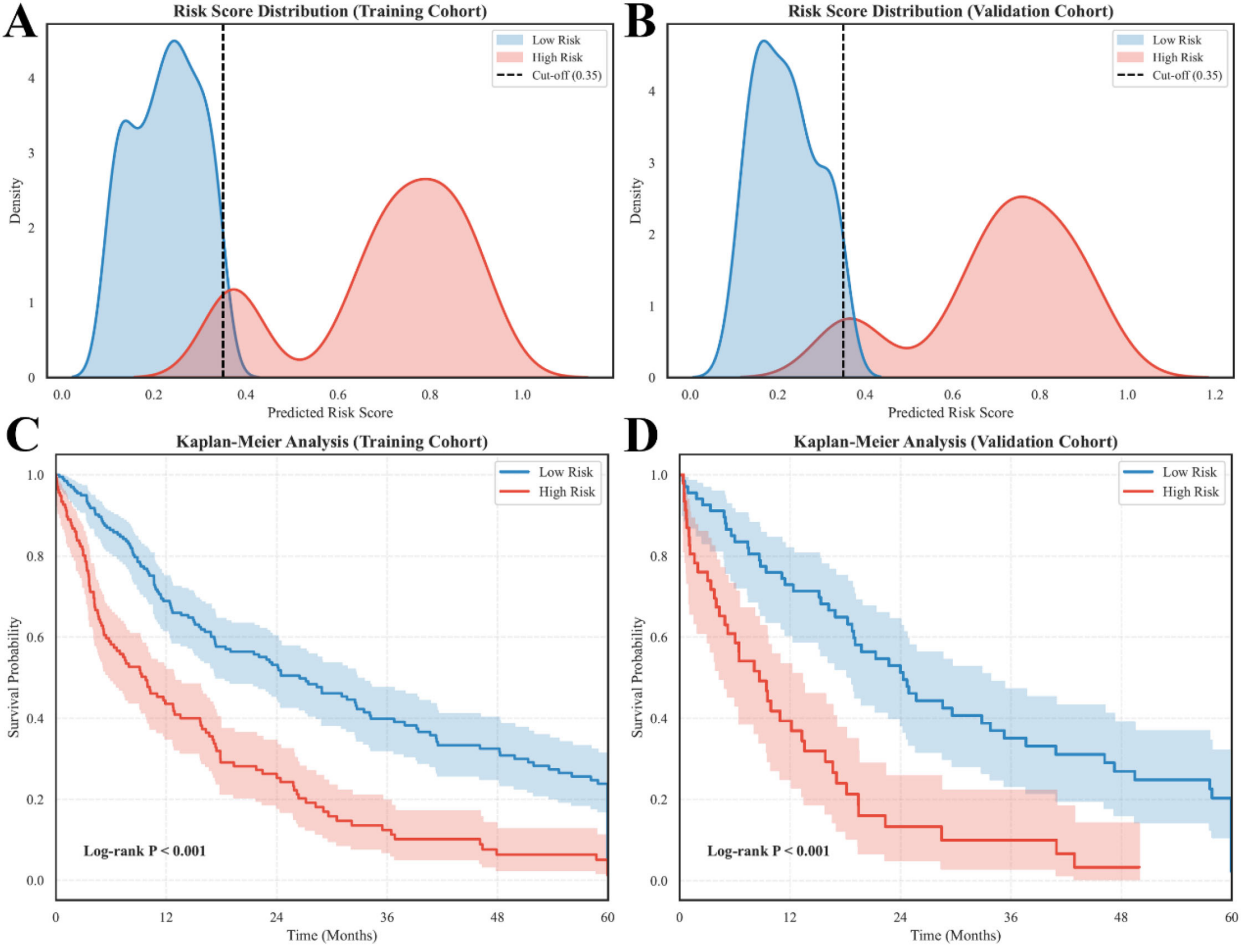
Risk stratification and survival analysis. **(A, B)** Distribution of predicted risk scores in the training and validation cohorts with the optimal cut-off value. **(C, D)** Kaplan-Meier survival curves comparing overall survival between low-risk and high-risk groups.

We performed prognostic stratification based on the predicted risk scores to facilitate the clinical application of the TabNet-Cox model. The distribution of risk scores in both the training (**Figure 3A**) and validation (**Figure 3B**) cohorts presented a clear bimodal pattern. This distribution indicates robust discrimination between patients with distinct prognoses. We categorized patients into low-risk and high-risk groups using an optimal cut-off value of 0.35. Survival analysis confirmed the validity of this stratification. The high-risk group exhibited significantly inferior overall survival compared to the low-risk group as shown in **Figures 3C, D** (Training: *χ^2^ = 33.4, P < 0.001*; Validation: *χ^2^ = 21.9, P < 0.001*). The survival curves showed significant and sustained divergence.

**Figure 4:**
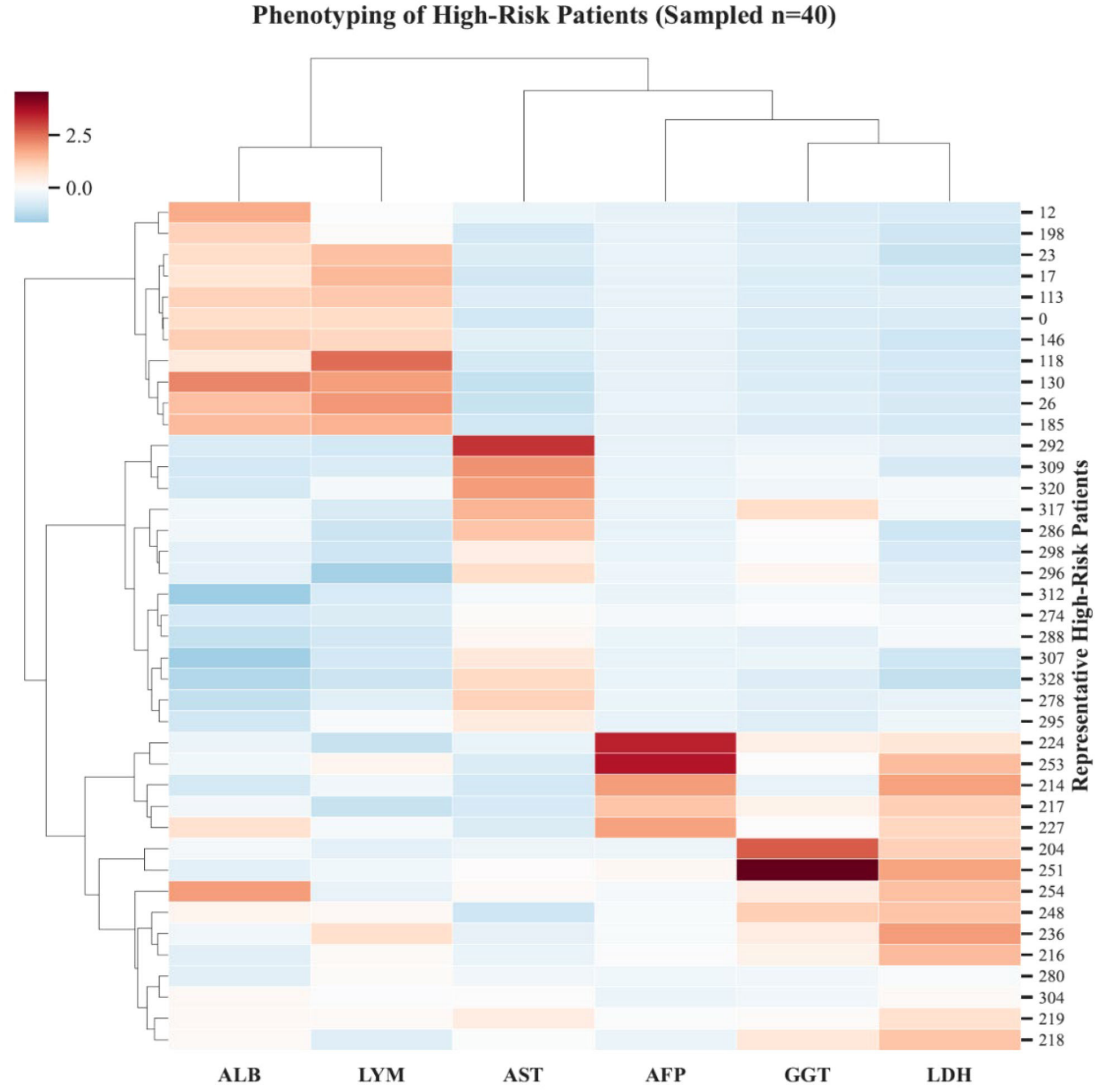
Identification of high-risk clinical profiles.

We conducted an unsupervised clustering analysis to investigate the underlying clinical drivers within the high-risk population (**Figure 4**). The heatmap revealed distinct clinical-pathological profiles among high-risk patients. One subgroup was characterized by concurrent elevations in AFP, GGT, and LDH. This profile suggests a “tumor burden-dominant” pattern. Another subgroup exhibited prominent AST elevation combined with hypoalbuminemia. This profile aligns with a “liver dysfunction-dominant” pattern. These findings indicate that the TabNet-Cox model captures diverse pathophysiological mechanisms including aggressive tumor behavior and hepatic decompensation that contribute to poor therapeutic outcomes in ICI-treated HCC.

### 3.6 Model calibration and clinical utility

**Figure 5:**
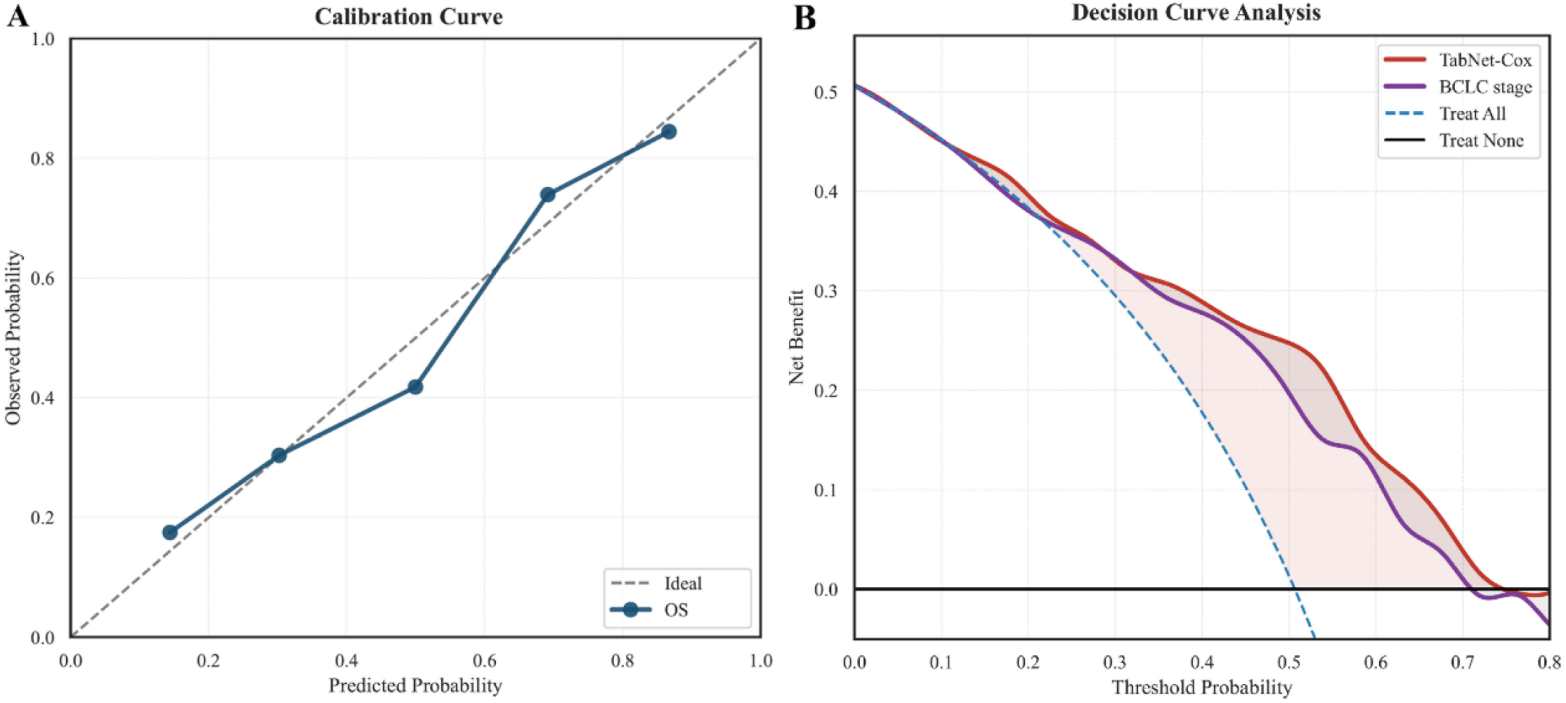
Calibration curve and DCA in the validation cohort. **(A)** Calibration curve showing agreement between predicted and observed survival probabilities. **(B)** Decision curve analysis demonstrating the net clinical benefit of the TabNet-Cox model across threshold probabilities.

Calibration analysis and DCA were performed in the validation cohort to assess the reliability and clinical usefulness of the TabNet-Cox model. The calibration curve (**Figure 5A**) demonstrated good agreement between predicted and observed survival probabilities across the full range, indicating accurate risk estimation within the validation set. The DCA (**Figure 5B**) showed that TabNet-Cox achieved a higher net benefit than both the treat-all and treat-no strategies across a wide range of threshold probabilities. To enable a fair comparison with routine clinical practice, the BCLC reference curve was constructed by generating time-specific risk estimates from a BCLC-only model using the same evaluation horizon. When compared with the current standard reference (BCLC stage), TabNet-Cox also demonstrated an incremental net benefit over a clinically relevant range, with the most evident advantage approximately between 0.35 and 0.70. These findings support the potential clinical utility of TabNet-Cox for risk-informed decision-making in patients with advanced hepatocellular carcinoma.

### 3.7 External validation in an independent cohort

**Figure 6:**
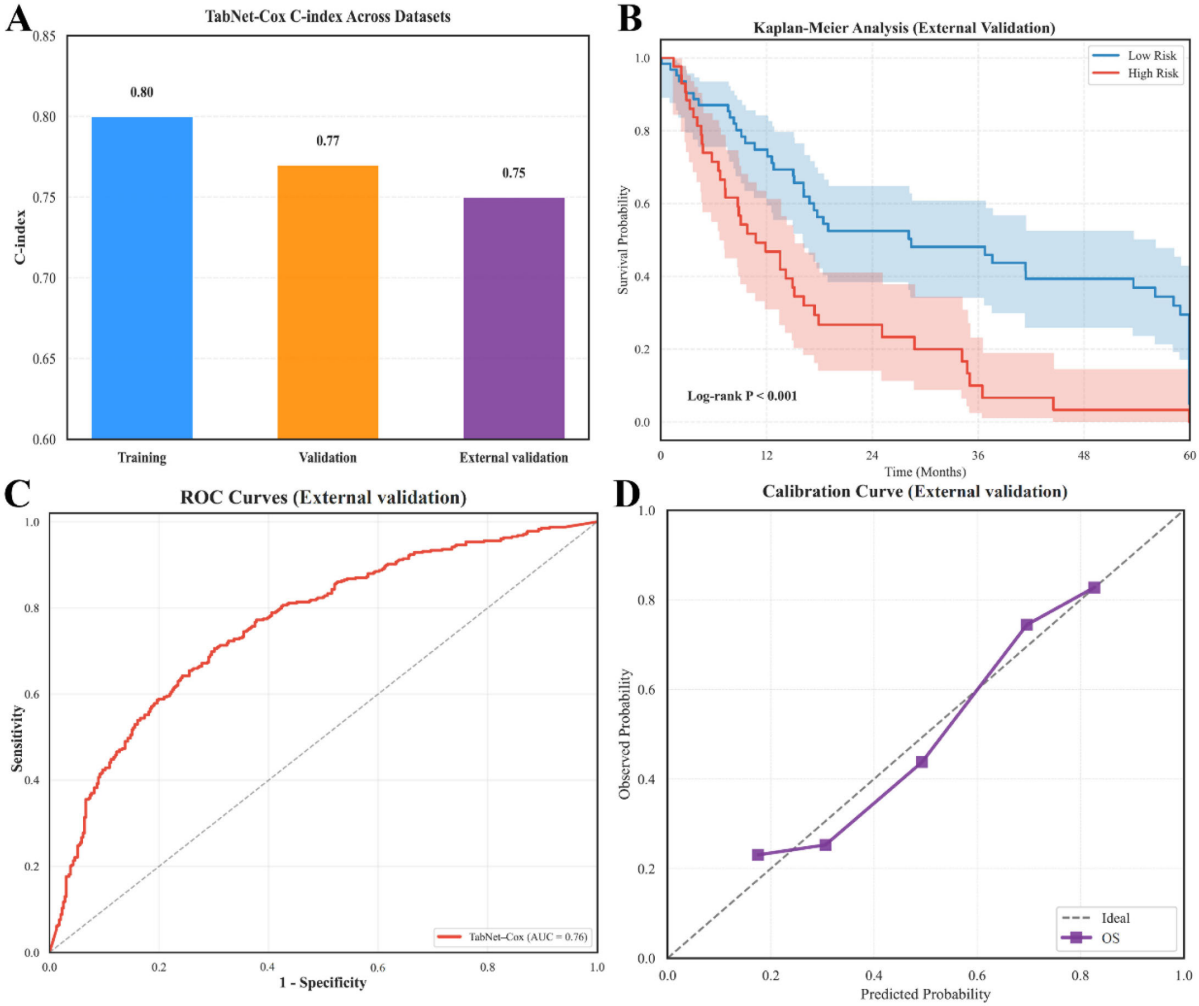
External validation of the TabNet-Cox model. **(A)** C-index across training, validation, and external validation cohort. **(B)** Kaplan–Meier curves for low- and high-risk groups in the external validation cohort. **(C)** ROC curve in the external validation cohort. **(D)** Calibration curve in the external validation cohort.

To further evaluate the generalizability of the TabNet-Cox model, we validated the trained model in an independent external cohort. Baseline characteristics were broadly comparable across the training, internal validation, and external cohorts, with no statistically significant differences observed in key clinical variables; detailed information is provided in **Supplementary Table 2** (all *P > 0.05*). As shown in **Figure 6A**, the discrimination performance remained robust across datasets, with the C-index decreasing only modestly from 0.80 in the training cohort and 0.77 in the internal validation cohort to 0.75 in the external validation cohort. Using the pre-specified risk score cut-off derived from the development cohort, TabNet-Cox preserved its risk-stratification capability in the external cohort. Kaplan–Meier analysis demonstrated clear and sustained separation between the high- and low-risk groups, with significantly worse overall survival observed in the high-risk group (*χ² = 6.4, P < 0.001*; **Figure 6B**). Consistently, the ROC analysis in the external cohort showed good discriminatory ability, yielding an AUC of 0.76 (**Figure 6C**). Calibration assessment further indicated acceptable agreement between predicted and observed survival probabilities in the external cohort, with the calibration curve showing overall proximity to the ideal diagonal line (**Figure 6D**).

### 3.8 Independent prognostic factors

**Figure 7:**
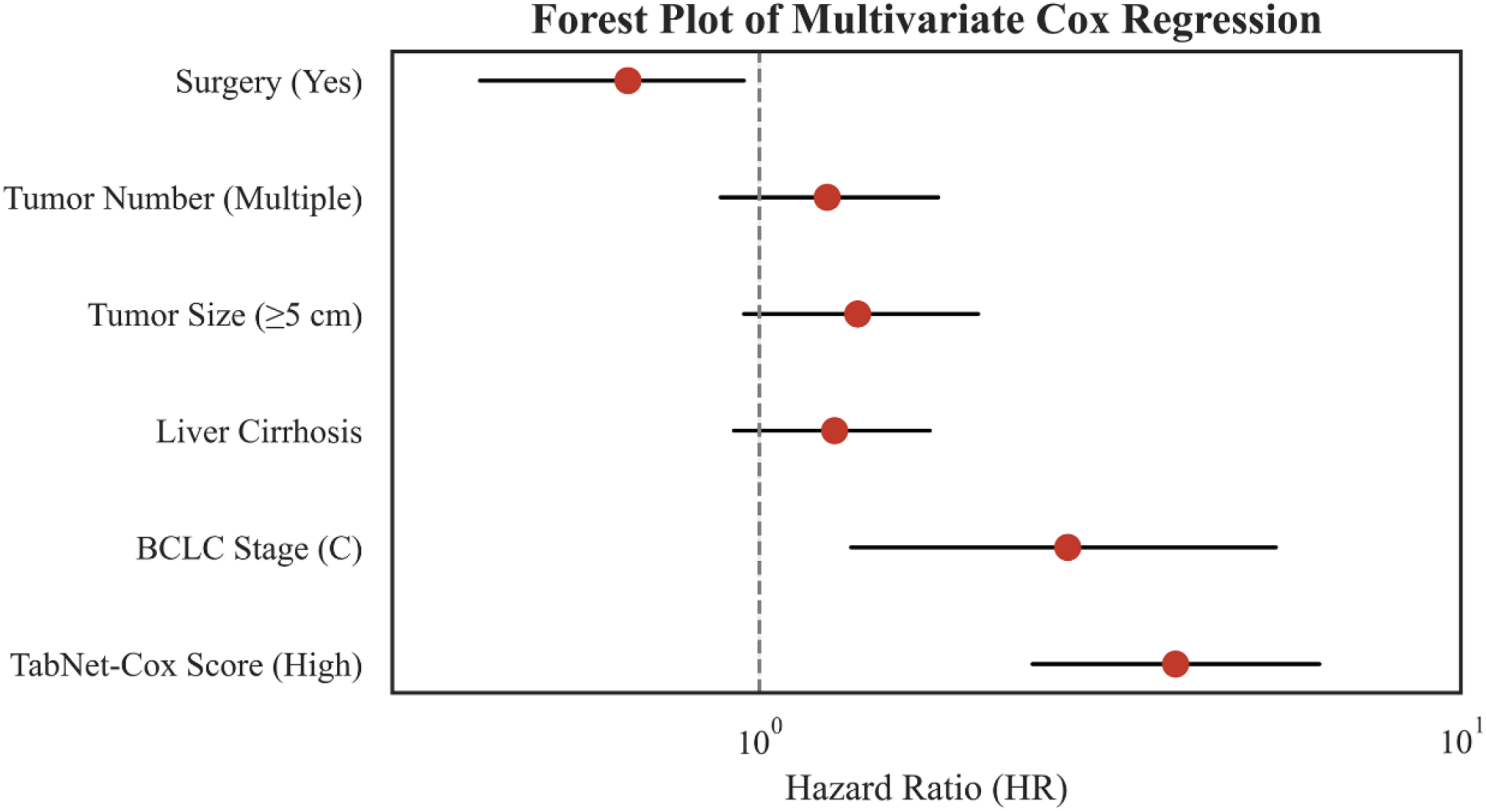
Multivariate Cox regression forest plot.

Cox regression analyses were performed to identify independent prognostic factors associated with overall survival (**Table 4**). In the univariate analysis, surgery, tumor number, tumor size, liver cirrhosis, BCLC stage, and the TabNet-Cox risk score were significantly associated with patient survival (all *P < 0.01*). In the multivariate Cox model, surgery remained an independent protective factor (*HR = 0.65, 95% CI: 0.40-0.95, P = 0.035*). BCLC stage C was independently associated with markedly worse survival compared with stages A-B (*HR = 2.75, 95% CI: 1.35-5.45, P < 0.001*). Notably, the TabNet-Cox score demonstrated the strongest prognostic value, with high-score patients exhibiting significantly increased mortality risk (*HR = 3.92, 95% CI: 2.45-6.28, P < 0.001*). These findings suggest that, within this cohort of patients receiving ICIs, the TabNet-Cox risk score provides additional prognostic insight beyond conventional clinicopathological variables and may serve as an important independent indicator of OS (**Figure 7**).

**Table 4:** Cox regression analyses in the validation cohort.

Variables	Univariate analysis	P	Multivariate analysis	P
	HR (95% CI)		HR (95% CI)	
Age (years)	1.01 (0.99–1.03)	0.452		
Sex (Male *vs*. Female)	1.15 (0.82–1.60)	0.388		
BMI (kg/m²)	0.98 (0.92–1.04)	0.615		
Smoking (Yes *vs*. No)	1.08 (0.75–1.55)	0.672		
Drinking (Yes *vs*. No)	1.12 (0.70–1.78)	0.620		
Surgery (Yes *vs*. No)	0.42 (0.28–0.65)	<0.001	0.65 (0.40–0.95)	0.035
Tumor Number (Multiple *vs*. Single)	1.65 (1.20–2.25)	0.002	1.25 (0.88–1.80)	0.185
Tumor Size (≥5 cm *vs*. <5 cm)	2.10 (1.45–3.05)	<0.001	1.38 (0.95–2.05)	0.085
Liver Cirrhosis (Yes *vs*. No)	1.55 (1.12–2.15)	0.008	1.28 (0.92–1.75)	0.120
BCLC Stage (C *vs*. A+B)	3.37 (2.15–5.95)	<0.001	2.75 (1.35–5.45)	<0.001
TabNet-Cox Score (High *vs*. Low)	4.25 (2.70–6.25)	<0.001	3.92 (2.45–6.28)	<0.001

### 3.9 Subgroup survival analysis

**Figure 8:**
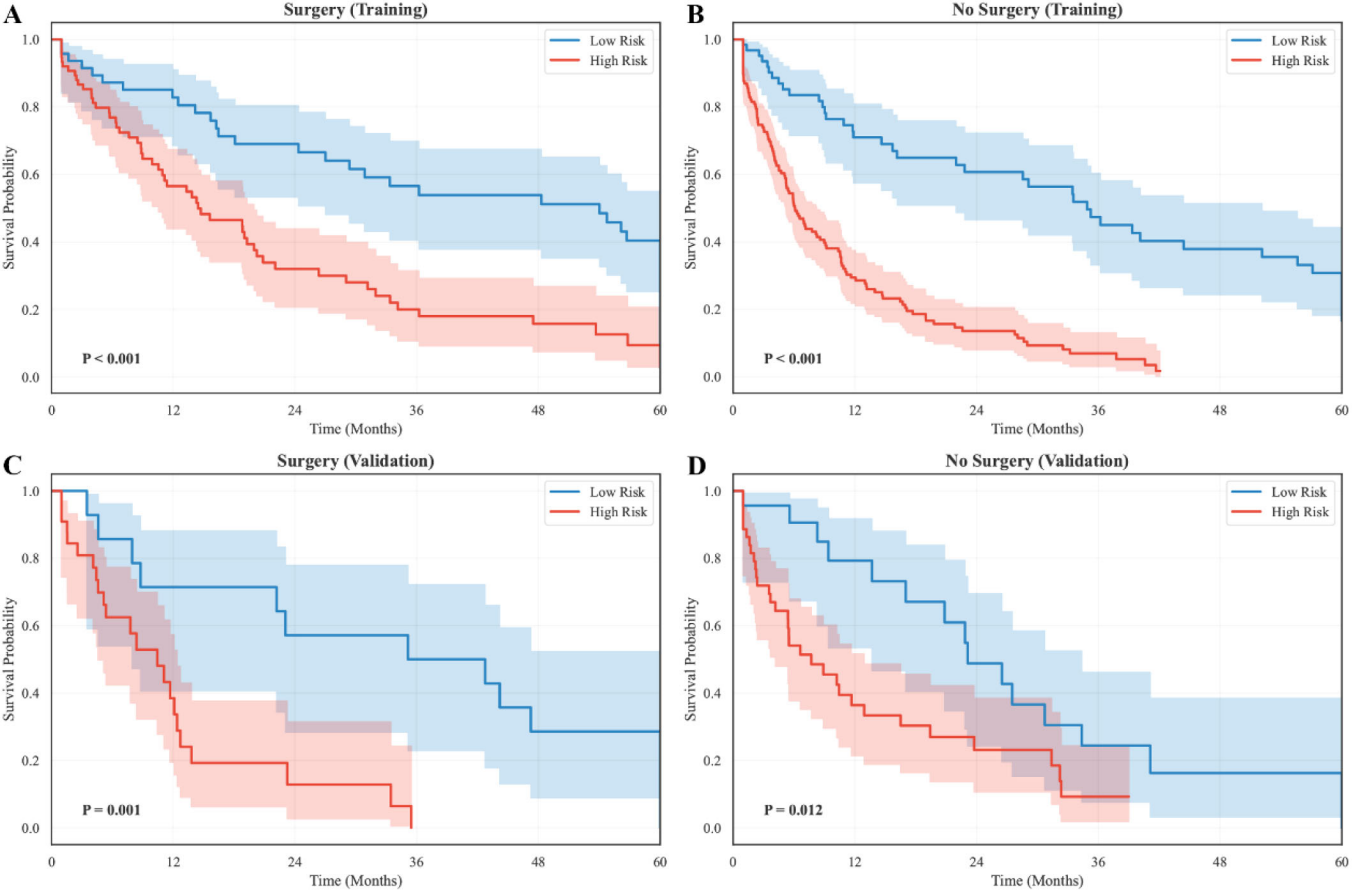
Subgroup survival analysis by surgery status. **(A)** Kaplan-Meier survival curves for low- and high-risk patients who underwent surgery in the training cohort. **(B)** Survival curves for patients without surgery in the training cohort. **(C)** Survival curves for surgical patients in the validation cohort. **(D)** Survival curves for non-surgical patients in the validation cohort.

To further assess the robustness of the TabNet-Cox model across clinically meaningful patient subpopulations, subgroup survival analyses were conducted based on the independent prognostic variables identified in the multivariate Cox model, namely surgery status and BCLC stage. Patients were divided into high- and low-risk groups using the same optimal cut-off (0.35), and survival differences were evaluated using Kaplan–Meier analysis. In the surgery subgroups, the model maintained clear discriminative ability. In the training cohort, both the surgery and no-surgery groups showed significant and sustained separation between risk strata (**Figures 8A, B**; Surgery: *χ² = 18.6, P < 0.001*; No Surgery: *χ² = 27.4, P < 0.001*). In the validation cohort, although the sample size was smaller, the model continued to identify distinct risk profiles with meaningful survival differences (**Figures 8C, D**; Surgery: *χ² = 9.8, P = 0.001*; No Surgery: *χ² = 6.4, P = 0.012*). Across all subgroups, high-risk patients consistently exhibited markedly inferior survival, demonstrating the model’s stability regardless of surgical treatment status.

**Figure 9:**
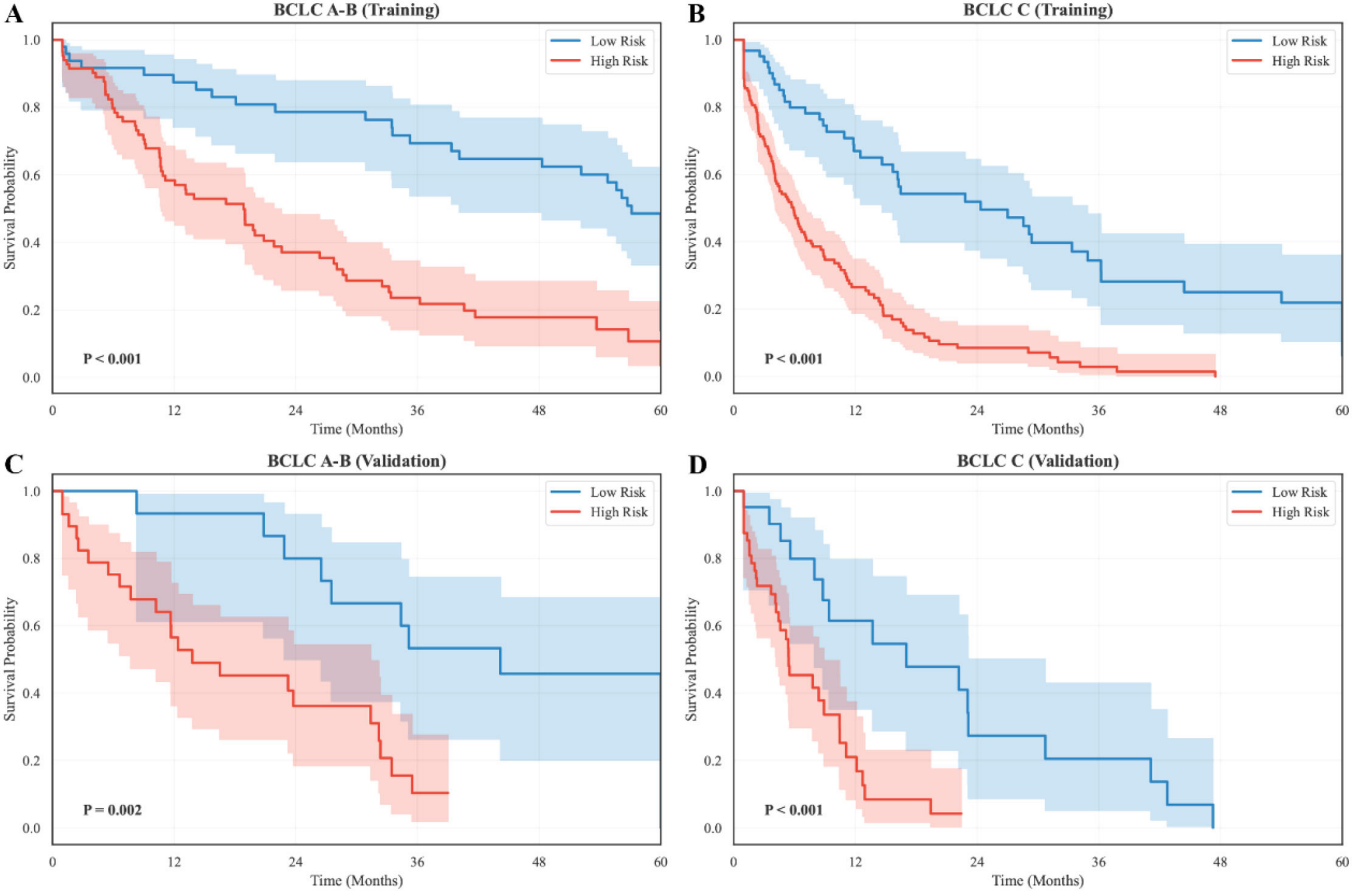
Subgroup survival analysis by BCLC stage. **(A)** Kaplan-Meier survival curves for BCLC A-B patients in the training cohort. **(B)** Survival curves for BCLC C patients in the training cohort. **(C)** Survival curves for BCLC A-B patients in the validation cohort. **(D)** Survival curves for BCLC C patients in the validation cohort.

Similarly, stratification by BCLC stage showed preserved discriminatory performance. In the training cohort, significant divergence was observed in both early-intermediate (A-B) and advanced (C) stages (**Figures 9A, B**; A-B: *χ² = 22.7, P < 0.001*; C: *χ² = 31.5, P < 0.001*). In the validation cohort, the separation between low- and high-risk groups remained statistically meaningful despite smaller sample numbers (**Figures 9C, D**; A-B: *χ² = 8.9, P = 0.002*; C: *χ² = 12.4, P < 0.001*). As expected, patients with BCLC C showed worse overall outcomes than those with A–B disease, yet risk stratification remained effective within each clinical stratum.

## 4 Discussion

ICIs have revolutionized the therapeutic landscape for HCC, yet the marked heterogeneity in treatment response underscores the urgent need for precise prognostic tools (26). In this study, we developed and validated an interpretable deep learning model, TabNet-Cox, designed to predict overall survival in HCC patients receiving ICIs. By integrating multidimensional clinical features, the TabNet-Cox model demonstrated superior predictive performance compared with traditional Cox regression and other machine-learning algorithms. Furthermore, we decoded the model’s “black box” using SHAP values and attention mechanisms, which revealed distinct high-risk phenotypes characterized by elevated tumor burden and hepatic dysfunction.

Our findings highlight the advantage of deep learning architectures specifically tailored for tabular data in medical prognosis. The TabNet-Cox model achieved the highest C-index of 0.79 and the lowest Brier score among all tested models. Although the conventional Cox proportional hazards model is widely used, it depends on the assumption of linear associations between covariates and risk, which often oversimplifies the complex biological interactions present in cancer (27, 28). Standard deep learning models such as DeepSurv can capture nonlinear relationships, but they typically lack transparency (25, 29, 30). TabNet addresses these limitations by employing a sequential attention mechanism that not only mimics the decision-making process of decision trees but also preserves the representation-learning strengths of deep neural networks (31). This architecture enables the model to focus on the most salient features at each decision step, effectively filtering out noise and enhancing generalization (32). The consistent performance observed between our training and validation cohorts further supports this improvement.

Feature importance analysis revealed biologically plausible predictions that align closely with established HCC pathophysiology. AFP emerged as the most critical prognostic factor, followed by GGT, LDH, and lymphocyte count. AFP is a well-recognized marker of tumor burden and biological aggressiveness in HCC (33). Elevated GGT and LDH levels are frequently associated with oxidative stress, tumor necrosis, and a hypoxic microenvironment—conditions that can promote immunosuppression and confer resistance to ICIs (34–36). Conversely, higher lymphocyte counts and albumin levels were identified as protective factors (37). As the primary effectors of anti-tumor immunity, lymphocytes contribute to treatment effectiveness, and a robust baseline lymphocyte reservoir is typically linked to a stronger response to PD-1/PD-L1 blockade (38, 39). By capturing the interplay between tumor aggressiveness and host immune reserve, the model provides a holistic assessment of a patient’s overall status.

A unique contribution of this study is the identification of distinct clinical–pathological profiles within the model-defined high-risk population. Using unsupervised clustering, we observed two divergent patterns associated with poor prognosis. The first is a “tumor burden-dominant” subtype, characterized by higher AFP and LDH levels, whereas the second is a “liver dysfunction-dominant” subtype, marked by elevated AST and lower albumin. This distinction is clinically intuitive and helps contextualize heterogeneity among high-risk patients. Conceptually, the former pattern suggests a prognosis more strongly driven by tumor burden, while the latter appears more constrained by limited hepatic reserve. Accordingly, these phenotypes may serve as a hypothesis-generating interpretability layer to motivate future studies evaluating phenotype-stratified treatment strategies (e.g., regimen intensification or integration of locoregional approaches in tumor-burden–dominant cases, versus liver function–preserving and toxicity-mitigating strategies in liver-dysfunction–dominant cases) (40–43). Overall, this phenotype-level characterization complements risk scoring by providing an interpretable framework to describe high-risk heterogeneity and to support prospective validation and treatment–phenotype interaction testing.

Subgroup analyses confirmed the robustness of the TabNet-Cox model, as it maintained excellent discriminatory ability across different BCLC stages and treatment modalities. Notably, the model effectively distinguished patients with poor outcomes even within early and intermediate-stage HCC, indicating that substantial biological heterogeneity exists within the same clinical stage. Decision Curve Analysis further reinforced the clinical utility of the model, demonstrating a consistently higher net benefit than both “treat-all” and “treat-none” strategies across a broad range of threshold probabilities. Incorporating TabNet-Cox into risk stratification could enable oncologists to identify patients who may benefit from intensified monitoring and potentially guide the selection of alternative therapeutic approaches, all without increasing the burden of overtreatment.

This study has several limitations. First, selection bias is inevitable due to the retrospective design, and the findings may reflect the patient spectrum and treatment practices of the participating institutions. Second, although we incorporated an independent external validation cohort and used resampling-based evaluation to reduce the risk of overfitting, all cohorts were retrospective and derived from a limited number of centers. Therefore, broader multicenter prospective validation with more diverse populations is still warranted to further establish generalizability. Third, treatment allocation was not randomized in this real-world setting, which limits causal inference regarding regimen-specific benefit and constrains our ability to translate the identified phenotypes into treatment-directive recommendations. Accordingly, these phenotypes should be interpreted as hypothesis-generating. In addition, patients had heterogeneous treatment histories prior to ICI initiation. Although we have now supplemented detailed information on prior surgery and locoregional therapies (including TACE, HAIC, radiotherapy, and ablation) and confirmed that these treatments were comparable between cohorts, prior treatment exposure and subsequent post-baseline management cannot be fully standardized or completely controlled in a retrospective real-world dataset. As a result, residual confounding related to treatment sequencing and intensity may still exist. Fourth, although TabNet-Cox was developed to predict overall survival under ICI-based therapy, this study was not specifically designed to directly evaluate immunotherapy efficacy or early treatment response. In retrospective cohorts, ICI efficacy can be defined by multiple endpoints (e.g., ORR, DCR, PFS, or durable clinical benefit), which are subject to heterogeneity in assessment timing, imaging availability, and downstream therapies. While the baseline risk score generated by TabNet-Cox may hold potential as a candidate biomarker associated with treatment outcomes, its role as an early predictor of ICI efficacy should be considered exploratory and requires dedicated prospective validation with standardized response assessments. Finally, although we included a comprehensive set of routinely available clinical and laboratory variables, incorporating established immunotherapy biomarkers or multi-modal data, such as PD-L1 status, tumor mutational burden, ctDNA dynamics, or radiomic features, could further enhance predictive performance and clinical utility. Overall, the findings of this study should be further validated in large-scale, multicenter prospective cohorts with standardized treatment and follow-up protocols.

## 5 Conclusion

In conclusion, we developed a robust and interpretable TabNet-Cox model to predict survival in HCC patients receiving ICIs. By integrating multidimensional clinical features, this model provides a reliable tool for individualized risk stratification. Moreover, the identification of key prognostic factors and distinct high-risk phenotypes offers valuable biological insights, thereby supporting the development of more precise and personalized therapeutic strategies in HCC management.

## Data Availability

The raw data supporting the conclusions of this article will be made available by the authors, without undue reservation.
