# Supplementary material for: An interpretable deep learning framework based on TabNet-Cox for risk stratification and prognostic assessment in hepatocellular carcinoma immunotherapy
Source: Front Immunol. 2026 Feb 11;17:1751829. doi: 10.3389/fimmu.2026.1751829 (PMC12932533; doi:10.3389/fimmu.2026.1751829)
Supplement: Supplementary file 1 [file Table1.docx]

**Supplementary Table 1** Hyperparameter Search Space for TabNet-Cox

| **Hyperparameter** | **Search Range / Candidate Values** | **Final Value** |
| --- | --- | --- |
| *n*_d_, *n*_a_ | 8, 16, 32, 64 | 16 |
| *N*_steps_ | 3, 4, 5 | 4 |
| γ | 1.2, 1.5, 2.0 | 1.5 |
| λ_sparse_ | 1 × 10^−3^, 1 × 10^−4^, 1 × 10^−5^ | 1 × 10^−4^ |

**Supplementary Table 2** Baseline characteristics and comparability across cohorts.

| **Items** | Training (n=339) | Validation (n=114) | External Validation (n=105) | P value |
| --- | --- | --- | --- | --- |
| **Sex** |  |  |  | 0.652 |
| Male | 277 (81.7%) | 95 (83.3%) | 79 (75.2%) |  |
| Female | 62 (18.3%) | 19 (16.7%) | 26 (24.8%) |  |
| **Age (years)** | 57.53 ± 9.20 | 57.07 ± 8.89 | 58.23 ± 9.10 | 0.67 |
| **BMI (Kg/m²)** | 23.28 ± 3.49 | 23.53 ± 3.76 | 23.01 ± 3.88 | 0.333 |
| **ALBI** | -2.41 ± 0.44 | -2.63 ± 0.38 | -2.55 ± 0.45 | 0.665 |
| **Smoking** |  |  |  | 0.381 |
| Yes | 69 (20.4%) | 24 (21.1%) | 29 (27.6%) |  |
| No | 270 (79.6%) | 90 (78.9%) | 76 (72.4%) |  |
| **Drinking** |  |  |  | 0.515 |
| Yes | 41 (12.1%) | 16 (14.0%) | 17 (16.2%) |  |
| No | 298 (87.9%) | 98 (86.0%) | 88 (83.8%) |  |
| **ABO blood type** |  |  |  | 0.634 |
| A | 92 (27.1%) | 34 (29.8%) | 32 (30.5%) |  |
| B | 104 (30.7%) | 31 (27.2%) | 28 (26.7%) |  |
| AB | 53 (15.6%) | 13 (11.4%) | 12 (11.4%) |  |
| O | 90 (26.5%) | 36 (31.6%) | 33 (31.4%) |  |
| **Treatment** |  |  |  |  |
| Surgery | 112 (33.0%) | 41 (36.0%) | 39 (37.1%) | 0.343 |
| Locoregional therapy | 199 (58.7%) | 64 (56.1%) | 56 (53.3%) |  |
| None | 28 (8.3%) | 9 (7.9%) | 10 (9.6%) |  |
| **Tumor number** |  |  |  | 0.713 |
| Single | 137 (40.4%) | 49 (43.0%) | 40 (38.1%) |  |
| Multiple | 202 (59.6%) | 65 (57.0%) | 65 (61.9%) |  |
| **Tumor size** |  |  |  | 0.475 |
| <5 cm | 59 (17.4%) | 25 (21.9%) | 22 (21.0%) |  |
| ≥5 cm | 280 (82.6%) | 89 (78.1%) | 83 (79.0%) |  |
| **Liver cirrhosis** |  |  |  | 0.742 |
| Yes | 100 (29.5%) | 35 (30.7%) | 30 (28.6%) |  |
| No | 239 (70.5%) | 79 (69.3%) | 75 (71.4%) |  |
| **BCLC stage** |  |  |  | 0.814 |
| A + B | 147 (43.4%) | 51 (44.7%) | 43 (41.0%) |  |
| C | 192 (56.6%) | 63 (55.3%) | 62 (59.0%) |  |

**Supplementary Table 3** Abbreviation list

| **Abbreviation** | **Full term** |
| --- | --- |
| AFP | Alpha-fetoprotein |
| ALB | Albumin |
| ALBI | Albumin–bilirubin |
| ALP | Alkaline phosphatase |
| ALT | Alanine aminotransferase |
| AST | Aspartate aminotransferase |
| AUC | Area under the curve |
| BCLC | Barcelona Clinic Liver Cancer |
| BMI | Body mass index |
| CA19-9 | Carbohydrate antigen 19-9 |
| CEA | Carcinoembryonic antigen |
| CI | Confidence interval |
| C-index | Concordance index |
| Cox | Cox proportional hazards model |
| CREA | Creatinine |
| CSCO | Chinese Society of Clinical Oncology |
| ctDNA | Circulating tumor DNA |
| DBIL | Direct bilirubin |
| DCA | Decision curve analysis |
| DL | Deep learning |
| Fbg | Fibrinogen |
| GBSA | Gradient-boosting survival analysis |
| GGT | Gamma-glutamyl transferase |
| Glu | Glucose |
| GLOB | Globulin |
| HCC | Hepatocellular carcinoma |
| HCT | Hematocrit |
| HGB | Hemoglobin |
| HR | Hazard ratio |
| IBS | Integrated Brier score |
| ICIs | Immune checkpoint inhibitors |
| IDBIL | Indirect bilirubin |
| IgA | Immunoglobulin A |
| IgG | Immunoglobulin G |
| IgM | Immunoglobulin M |
| INR | International normalized ratio |
| IQR | Interquartile range |
| KNN | k-nearest neighbors |
| LDH | Lactate dehydrogenase |
| LYM | Lymphocyte count |
| ML | Machine learning |
| MON | Monocyte count |
| NEU | Neutrophil count |
| OS | Overall survival |
| PALB | Prealbumin |
| PD-1 | Programmed cell death protein 1 |
| PD-L1 | Programmed death-ligand 1 |
| PLT | Platelet count |
| PT | Prothrombin time |
| RBC | Red blood cell count |
| ROC | Receiver operating characteristic |
| RSF | Random survival forest |
| SD | Standard deviation |
| SHAP | SHapley Additive exPlanations |
| TabNet | Tabular neural network |
| TBIL | Total bilirubin |
| TMB | Tumor mutational burden |
| TKIs | Tyrosine kinase inhibitors |
| TP | Total protein |
| TT | Thrombin time |
| UA | Uric acid |
| VIF | Variance inflation factor |
| WBC | White blood cell count |
