# Supplementary figures and images for: An interpretable deep learning framework based on TabNet-Cox for risk stratification and prognostic assessment in hepatocellular carcinoma immunotherapy
Source: Front Immunol. 2026 Feb 11;17:1751829. doi: 10.3389/fimmu.2026.1751829 (PMC12932533; doi:10.3389/fimmu.2026.1751829)

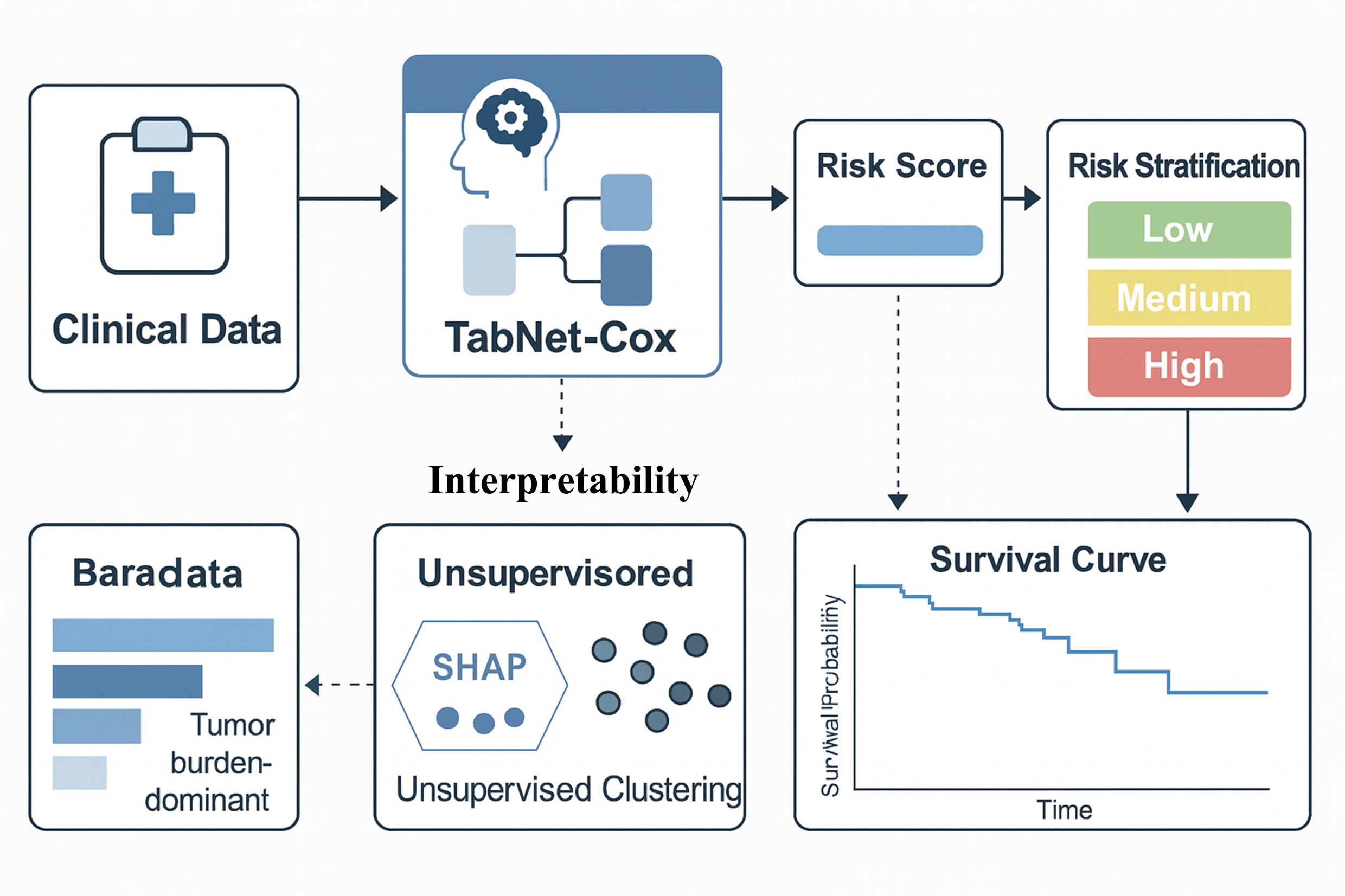

Supplement: Supplementary file 2 [file Image1.tif]
